# Dealing With the COVID-19 Infodemic: Distress by Information, Information Avoidance, and Compliance With Preventive Measures

**DOI:** 10.3389/fpsyg.2020.567905

**Published:** 2020-11-05

**Authors:** Katharina U. Siebenhaar, Anja K. Köther, Georg W. Alpers

**Affiliations:** Department of Psychology, School of Social Sciences, University of Mannheim, Mannheim, Germany

**Keywords:** COVID-19, emotional distress, information avoidance, eHealth literacy, trust in media, compliance

## Abstract

In the ongoing coronavirus disease 2019 (COVID-19) pandemic, media reports have caused anxiety and distress in many. In some individuals, feeling distressed by information may lead to avoidance of information, which has been shown to undermine compliance with preventive health behaviors in many health domains (e.g., cancer screenings). We set out to examine whether feeling distressed by information predicts higher avoidance of information about COVID-19 (avoidance hypothesis), and whether this, in turn, predicts worse compliance with measures intended to prevent the spread of COVID-19 (compliance hypothesis). Thus, we conducted an online survey with a convenience sample (*N* = 1,059, 79.4% female) and assessed distress by information, information avoidance, and compliance with preventive measures. Furthermore, we inquired about participants’ information seeking behavior and media usage, their trust in information sources, and level of eHealth literacy, as well as generalized anxiety. We conducted multiple linear regression analyses to predict distress by information, information avoidance, and compliance with preventive measures. Overall, distress by information was associated with better compliance. However, distress was also linked with an increased tendency to avoid information (avoidance hypothesis), and this reduced compliance with preventive measures (compliance hypothesis). Thus, distress may generally induce adaptive behavior in support of crisis management, unless individuals respond to it by avoiding information. These findings provide insights into the consequences of distress by information and avoidance of information during a global health crisis. These results underscore that avoiding information is a maladaptive response to distress by information, which may ultimately interfere with effective crisis management. Consequently, we emphasize the need to develop measures to counteract information avoidance.

## Introduction

In the coronavirus disease 2019 (COVID-19) pandemic, people have been exposed to an ongoing news cycle. This prompted the World Health Organization (WHO) to state that the healthcare system is not just fighting an epidemic, but also an infodemic. This refers to vast amounts of information that spread rapidly and can impede effective crisis management (Zarocostas, 2020). Thus, information is a mixed blessing in the COVID-19 pandemic. On the one hand, effective communication of facts helps individuals develop adequate risk perceptions and make adaptive health decisions to protect themselves and their peers (Garfin et al., 2020). On the other hand, vast amounts of information may also impose additional strain on crisis management (Kim et al., 2019; Garfin et al., 2020), as they trigger unpleasant emotions that can have undesired consequences (Sweeny et al., 2010).

Information and media coverage on content that is perceived as threatening can elicit aversive emotions, such as distress (Rubin et al., 2009; Wheaton et al., 2012; Pfefferbaum et al., 2014; Klemm et al., 2016; Thompson et al., 2017). When information is contradictory or uncertain, distress and risk perceptions may be even more elevated (Taha et al., 2014; Fischhoff et al., 2018). Past research on natural or human-made disasters showed that consuming more media coverage is typically associated with increased incidences of post-traumatic stress disorder (PTSD), anxiety, and depression (Pfefferbaum et al., 2014). More specific examples for media consumption and distress during viral outbreaks include the 2014 incidences of Ebola in the United States and the swine flu pandemic. Although individual risk was comparably low in both crises, media exposure to the topic was associated with heightened distress and functional impairment (e.g., Rubin et al., 2010; Wheaton et al., 2012; Thompson et al., 2017). Taking the high individual risk of the COVID-19 pandemic into account, it is not surprising that anxiety and distress have been elevated in response to the crisis (see Wang et al., 2020).

Besides adverse consequences for mental health, heightened distress by information can have relevant consequences for an individual’s behavior in the crisis (Jones and Salathe, 2009; Rubin et al., 2010; Bults et al., 2011). For example, during the swine flu pandemic, higher distress was associated with better compliance with preventive measures (Jones and Salathe, 2009; Rubin et al., 2009). Whereas this is clearly positive from a crisis management perspective, other consequences of distress may be undesirable. For instance, distress was also associated with increased utilization of healthcare services during past viral outbreaks, which put additional strain on already overburdened healthcare systems (McDonnell et al., 2012). Similarly, distress triggered panic purchases early in the COVID-19 pandemic. This led to global shortages of specific consumer goods and important medical equipment, such as hand sanitizer and face masks (Cheng et al., 2020; Garfin et al., 2020). Such behavioral consequences of distress may be most detrimental when they interfere with compliance with preventive measures. As it is not yet clear in what way distress influences compliance with preventive measures during the COVID-19 pandemic (Holmes et al., 2020), the examination of individuals’ responses to distressing health information is pertinent. In particular, responses that reduce compliance need to be identified so that authorities can adequately address them.

Individuals respond to threatening health information either by surveilling it and taking appropriate measures or by avoiding threatening information (Sweeny et al., 2010; Howell and Shepperd, 2013a, 2016). We focus on information avoidance, as this is one reaction to distressing information that has often been overlooked in previous research on responses to viral outbreaks. Findings from other health domains show that a substantial proportion of the population opts to avoid anxiety-provoking information, such as HIV status, cancer risk, or a genetic disposition to diseases (Hightow et al., 2003; Orom and Shepperd, 2015; Taber et al., 2015). Generally, health information avoidance is an emotionally driven, maladaptive defensive response (Howell and Shepperd, 2013b; Sweeny et al., 2010). According to the information avoidance framework, individuals most commonly avoid information when learning the information is associated with aversive emotions (e.g., receiving a cancer diagnosis elicits fear) or requires individuals to take undesired actions (e.g., undergoing surgery; Ajekigbe, 1991; Sweeny et al., 2010). Both responses are highly relevant in the case of COVID-19, as the topic not only is threatening but also requires individuals to take undesired actions (e.g., social distancing).

Furthermore, information avoidance can result from overexposure to health topics that receive an abundance of attention in the media (Barbour et al., 2012). In a recent survey, two thirds of participants reported feeling the need to take breaks from the news on COVID-19 (Mitchell et al., 2020). While this may help individuals remain calm, it also implies that they can miss out on important novel information (e.g., additional preventive measures, rising incidences in their area of residence) or may even underestimate the severity of the situation, no longer being confronted with it. Thereby, avoiding information about COVID-19 could result in intentional or unintentional worse compliance with preventive measures, with severe consequences for crisis management. In line with this, information avoidance has been associated with lower compliance to preventive behaviors in other health domains (Emanuel et al., 2015). However, to our knowledge, information avoidance and its potential consequences have not yet been assessed in a global health crisis.

We set out to examine whether distress caused by information about COVID-19, avoidance of information, and compliance with preventive measures in the case of COVID-19 are interrelated. We expected that a higher level of distress by information is associated with more avoidance of information (avoidance hypothesis), and that more avoidance of information is associated with worse compliance with preventive measures (compliance hypothesis).

In addition, we inquired about participants’ information seeking behavior, level of eHealth literacy, and trust in information sources. To date, individuals obtain news from a variety of sources, and some of these may be particularly at risk of spreading misinformation about COVID-19 (Depoux et al., 2020). Thus, individuals’ ability to find information and critically evaluate the reliability of information (i.e., eHealth literacy) may be decisive for their emotional and behavioral responses to this crisis (Sentell and Vamos, 2020). Moreover, considering information provided by health authorities and the media as trustworthy enhanced compliance with preventive measures during the swine flu pandemic (Rubin et al., 2009). Thus, we assumed that outlining the role of these variables and their interaction with distress by information, information avoidance, and compliance with preventive measures may aid the development of recommendations for action in the COVID-19 pandemic.

## Materials and Methods

### Participants

Participants were recruited from the community and via the social media platforms. Ethical approval for the study was granted by the ethics committee of the University of Mannheim. Initially, 1,432 participants started the online study. However, 26.05% dropped out before completing all questions, which is comparable to dropout rates reported in other online studies (Galesic, 2006; Hengen and Alpers, 2019). The majority of dropouts occurred directly after accessing the survey. All incomplete datasets were excluded. This resulted in a final sample of *N* = 1,059 participants (age; *M* = 39.53, *SD* = 12.85, 79.4% female, 44.4% university degree) and included participants from all 16 German states. Furthermore, a substantial number of participants had a preexisting mental health condition (28.4%) or a physical health condition that put them at higher risk of a severe progression of COVID-19 (30.6%). Finally, 3.6% of our sample had been tested for COVID-19 and 1% tested positive.

### Data Collection and Procedure

Data were collected from March 27 until April 29. Notably, in Germany, the strict regulations to slow down the spread of COVID-19 (i.e., contact restrictions) started on March 22 and were first relaxed on April 20. The study was presented in SoSci Survey and hosted on the university’s secure server. The online link to the study was distributed on social media and advertised on the website of our university. Participants accessed the study by clicking on the link. Prior to participation, individuals received general information about the study topic and procedure and provided informed consent. Then, participants completed a questionnaire battery, taking approximately 20 min. To measure our main outcome variables, this battery included the distress by information subscale of the Cyberchondria Severity Scale—15 (CSS-15; Barke et al., 2016), one self-generated item on information overload, the adapted Information Avoidance Scale (Howell and Shepperd, 2016), and a self-generated scale to assess compliance with preventive measures during the crisis. Furthermore, the following measures were also assessed and considered as predictors in the regression analyses when they significantly correlated with the outcome: sociodemographic data, information seeking behavior and media usage, the eHealth Literacy Scale (eHEALS; Norman and Skinner, 2006), and the Generalized Anxiety Disorder—7 instrument (GAD-7; Spitzer et al., 2006). On all measures, participants were instructed to report on their emotions and behavior since the start of the COVID-19 pandemic. Participants received no compensation for participation.

### Main Outcomes

#### Distress by Information

Distress by information about COVID-19 was assessed with the distress by information subscale of the CSS-15, which has previously been validated in a representative German sample (Barke et al., 2016). This subscale assesses heightened distress after obtaining health information on a 5-point Likert scale (1 = “never” to 5 = “always”). We asked participants to specifically refer to information about COVID-19 instead of health information in general. Furthermore, we added an item to capture the magnitude of information and distress (“the amount of information about COVID-19 is getting to be too much”).

#### Information Avoidance

Avoidance of information was assessed using the adapted Information Avoidance Scale (Howell and Shepperd, 2016). This instrument has high internal consistency and convergent and discriminant validity and provides stable results across time and different sample populations. We again adapted this scale to measure avoidance of information about COVID-19. Participants responded to items on a 7-point Likert scale (1 = “strongly disagree” to 7 = “strongly agree”). Our German translation (translated and back-translated by two bilingual psychologists) can be obtained upon request.

#### Compliance With Preventive Measures

We assessed compliance with preventive measures during the crisis on 13 items, which we generated according to recommendations of the German Federal Centre for Health Education (BZgA, 2020). Assessed behaviors included (1) staying at home, (2) following recommended hygiene regulations (washing hands regularly, cough and sneeze etiquette), (3) keeping an appropriate distance to other people, (4) wearing a face mask, (5) having in-person social contact, (6) going to a park or playground, (7) going to the gym, (8) going to a party, (9) going to a restaurant, (10) taking a trip, (11) visiting family, (12) using public transportation, and (13) excessive purchases. Results from an exploratory factor analysis for this scale are reported in the Supplementary Material. Although internal consistency was weak, we kept all 13 items in our final index as all behaviors are highly relevant in the COVID-19 pandemic.

Although all assessments referred to the entire time of the ongoing pandemic, we reminded participants that this applied to their behavior as well. This was to clarify that we were also interested in compliance with these measures before they became mandatory. Participants responded to items by indicating whether they had shown “less,” “no change,” or “more” of each one of the relevant behaviors during the crisis. Similar to previous studies in the field (e.g., Jones and Salathe, 2009), we scored behavior in an index and allocated one point when participants reported having shown more of a preventive behavior (e.g., staying at home, following recommended hygiene regulations, wearing a face mask, keeping an appropriate distance to other people), or when participants reported having shown less of behavior that could spread the virus or burden the system (e.g., social contacts in person, taking a trip, visiting family, using public transport, excessive purchases, going to a park or playground, a gym, a party, or a restaurant). Consequently, higher scores on the index indicate better compliance with preventive measures.

### Additional Variables of Interest

We also assessed information seeking behavior and media usage, eHealth literacy, and generalized anxiety to test their associations with distress by information, information avoidance, and compliance.

#### Information Seeking Behavior and Media Usage

We assessed information seeking behavior and media usage by asking participants if (and for how long) they followed the news on COVID-19, whether their media consumption had increased since the start of the crisis, and whether they searched online for COVID-19-related mental or physical health information (e.g., how to stay mentally healthy during quarantine). We presented participants with a list of information sources, including news channels’ websites, internet search engines, social media (authorities’ channels), social media (user-generated content), public TV, private TV, health authorities, friends and family, primary care physicians, and the newspaper. We asked participants to indicate which sources they had used to obtain information about COVID-19. Next, we asked participants to rate how trustworthy they considered all sources to be on a 5-point Likert scale (1 = “not trustworthy” to 5 = “trustworthy”). Thus, participants also rated the trustworthiness of the sources they did not use.

#### eHealth Literacy

We assessed eHealth literacy with the eHEALS (Norman and Skinner, 2006). The eHEALS is a widely used scale that captures an individual’s perceived ability and comfort to access and apply online health information. We adapted all items to ask participants specifically about their eHealth literacy regarding COVID-19. Participants answered all items on a 5-point Likert scale (1 = “strongly disagree” to 5 = “strongly agree”).

#### Anxiety

The level of anxiety experienced since the start of the crisis was assessed with the GAD-7 (Spitzer et al., 2006). This instrument asks participants to indicate how often they felt impaired by a series of symptoms on a scale from 1 to 4 (1 = “never” to 4 = “almost every day”). We selected this measure as it is widely used and its validity has been demonstrated with a large German sample (Löwe et al., 2008).

### Statistical Analysis

Statistical analyses were run in IBM SPSS Statistics 27 (SPSS Inc., 2020) and PROCESS (Hayes, 2020). Prior to all analyses, assumptions (e.g., multicollinearity) were tested, and when violated, appropriate corrections were applied. Furthermore, we adjusted the significance levels according to Bonferroni–Holmes to correct for multiple tests.

Prior to hypothesis testing, we calculated descriptive data on information seeking behavior. Trust ratings of information sources were compared between participants who reported the use of a certain source and participants who did not use this source. Furthermore, we calculated the average trust rating of all information sources used by a participant and examined the role of this as a predictor in the subsequent analysis. This trust variable had four missing values, as four participants did not report to use any information source.

We linearly transformed sum scores of distress by information, information avoidance, eHealth literacy, generalized anxiety, and the average trust in information sources used to a range of 0–100 to enhance comparability. We log-transformed the compliance score as the data were not normally distributed (participants generally reported high compliance). Then, we calculated correlational analyses to examine if our main outcome variables (distress by information, information avoidance, and compliance) were significantly associated with one another. We also tested their association with other variables (e.g., sociodemographic data, generalized anxiety, date of data collection). We conducted group comparisons to see whether individuals with a mental or physical health condition or individuals who searched health information online differed in levels of distress, information avoidance, and compliance with preventive measures. Significant variables were included as predictors into subsequent regression analyses. The date of an individual’s participation had no effect on any of the outcome variables, and hence time was not considered in the subsequent analyses.

We ran a stepwise linear regression to explore which variables predict distress by information. For hypothesis testing, we conducted two more regression analyses. These tested the predictive value of distress by information about information avoidance (avoidance hypothesis) and information avoidance on compliance with preventive measures (compliance hypothesis).

Finally, we further explored the interrelatedness of distress by information, information avoidance, and compliance with preventive measures in a mediation analysis (Model 4) using PROCESS (Hayes, 2020). Thus, we tested whether avoidance of information (M) mediates an effect of distress by information (X) on compliance with preventive measures (Y). We controlled for sociodemographic variables, anxiety, and eHealth literacy in this analysis. Furthermore, we report standardized effects and coefficients in the results of this analysis.

## Results

### Descriptive Data on Information Seeking and Media Usage

Of our large and diverse sample, 67.1% indicated that they had been following the media coverage on the COVID-19 outbreak for more than 1 month, whereas 30.1% indicated following the news for less than 1 month, and 2.7% reported not following the news. Furthermore, 66% indicated that their media consumption in the COVID-19 outbreak was higher than their regular media consumption. Furthermore, 80.7% reported to have searched online for COVID-19-related physical health information, and 42.6% reported to have searched for COVID-19-related mental health information.

**FIGURE 1:**
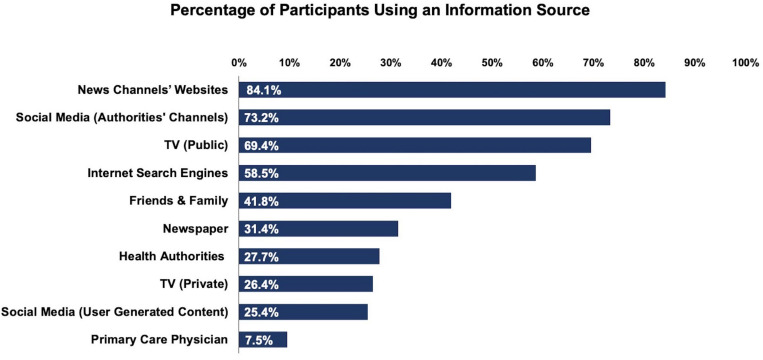
Percentage of participants reporting to use each source to obtain information about COVID-19.

Participants used a variety of information sources (*M* = 4.5, *SD* = 1.75), most of which were media sources (*M* = 3.68, *SD* = 1.46). Group comparisons showed that information sources were rated as more trustworthy by the participants who used them than by the participants who did not use them, *t*s ≥ 2.82, *p*s ≤ 0.004, *d*s ≥ 0.20. Exact statistical values are reported in the Supplementary Material. An overview of the information sources individuals used is provided in Figure 1.

### Correlations Among Main Outcomes and Other Variables

To test whether our main outcome variables were interrelated and to explore which other variables were associated with them, we first conducted correlational analyses. These showed that distress by information was associated with higher information avoidance, *r* = 0.269, *p* < 0.001, and higher information avoidance was associated with lower compliance with preventive measures, *r* = −0.146, *p* < 0.001. Thus, the requirements for our planned analyses were met. Interestingly, distress by information was also associated with higher compliance, *r* = 0.135, *p* < 0.001. We followed up on this effect after hypotheses testing in the mediation analysis below. These and all other correlations are presented in Table 1.

**TABLE 1:** Correlation analyses of distress by information, information avoidance, compliance with preventive measures, and other variables.

**Variables**		***N***	**(1)**	**(2)**	**(3)**	**(4)**	**(5)**	**(6)**
(1)	Distress by information	1,059						
(2)	Information avoidance	1,059	0.269**					
(3)	Compliance	1,059	0.135**	−0.146**				
(4)	eHealth literacy	1,059	−0.190**	−0.224**	0.083*			
(5)	Anxiety	1,059	0.531**	0.085**	0.078*	−0.098**		
(6)	Trust	1,055	–0.019	−0.222**	0.117**	0.290**	−0.103**	
(7)	Age	1,059	–0.042	−0.163**	0.138**	−0.077*	−0.075*	−0.015

*p < 0.05, two-tailed. **p < 0.01, two-tailed. In the correlations of distress by information, information avoidance, and compliance, we controlled for sociodemographic variables (age, gender, education), anxiety, and eHealth literacy.

### Regression on Distress by Information

We included variables that were significantly correlated with distress as predictors into a stepwise regression analysis. Furthermore, group comparisons showed that individuals with a preexisting mental or physical health condition and individuals searching online for physical or mental health information reported higher levels of distress, *t*s ≥ 2.8, *p*s ≤ 0.001, *d*s < 0.19. Thus, these variables were dummy coded and also entered into the analysis as predictors. The final model explained 33.9% variance of distress by information, model fit: *F*(6, 1,048) = 91.01, *p* < 0.001. Furthermore, results showed that higher generalized anxiety, β = 0.498, *t*(1,054) = 19.36, *p* < 0.001, lower eHealth literacy, β = −0.191, *t*(1,054) = −7.27, *p* < 0.001, searching physical health information online, β = 0.096, *t*(1,054) = 3.55, *p* < 0.001, searching mental health information online, β = 0.081, *t*(1,054) = 3.02, *p* = 0.003, trust in information sources used, β = 0.062, *t*(1,054) = 2.33, *p* = 0.020, and consuming more news than before the crisis, β = 0.056, *t*(1,054) = 2.18, *p* = 0.029, had incremental predictive value.

### Regression on Information Avoidance (Avoidance Hypothesis)

To test our avoidance hypothesis, we ran a regression on information avoidance with distress by information and other variables that correlated significantly with this outcome as predictors. Group comparisons showed no differences between participants with and without a preexisting physical health condition, *t*(1,057) = 1.43, *p* = 0.154, *d* = 0.09, but individuals with a preexisting mental health condition reported higher information avoidance than individuals without one, *t*(1,057) = 2.57, *p* = 0.01, *d* = 0.18. Thus, preexisting mental health condition was considered as a predictor in the analysis. Results supported our hypothesis, showing that higher distress by information was the most powerful predictor of higher information avoidance. The final model explained 18.3% of the variance, model fit: *F*(5, 1,049) = 48.31, *p* < 0.001, and other significant predictors of higher information avoidance included in the model were lower trust in information sources used, lower age, lower eHealth literacy, and lower generalized anxiety. Exact statistics are shown in Table 2.

**TABLE 2:** Summary of the final regression model on information avoidance.

**Step**	**Predictor**	**β**	**95% CI**		***t***	***p***	***R*^2^**	**Δ *R*^2^**
			**LL**	**UL**				
(1)	Distress by information	0.333	0.267	0.399	9.88	<0.001	0.088	
(2)	Trust	−0.195	−0.253	−0.138	−6.65	<0.001	0.134	0.046
(3)	Age	−0.171	−0.226	−0.116	−6.11	<0.001	0.157	0.023
(4)	eHealth literacy	−0.127	−0.186	−0.069	−4.26	<0.001	0.172	0.015
(5)	Anxiety	−0.132	−0.197	−0.067	−3.97	0.003	0.183	0.011

*n = 1,055. CI, confidence interval; LL, lower limit; UL, upper limit.*

### Regression on Compliance With Preventive Measures (Compliance Hypothesis)

To test our compliance hypothesis, avoidance of information and other variables significantly correlated with the outcome were entered into a stepwise regression model. Group comparisons showed that participants with a preexisting physical health condition and participants who previously searched for physical or mental health information online were more compliant, *t*s ≥ 2.99, *p*s ≤ 0.003, *d*s ≤ 0.19. Thus, these variables were included as predictors. Group comparisons regarding a preexisting mental health condition were non-significant, *t*(1,057) = 0.13, *p* = 0.896, *d* = 0.01. The final model explained 13.9% variance, model fit: *F*(8,1046) = 22.23, *p* < 0.001). Results supported our hypothesis, showing that lower avoidance of information was a significant predictor for better compliance with preventive measures. Other significant predictors of better compliance were searching online for physical health information, watching more news than before the crisis, higher age, higher education, more distress by information, a preexisting physical health condition, and female gender. Exact statistics are shown in Table 3.

**TABLE 3:** Summary of the final regression model on compliance with preventive measures.

	**Predictor**	**β**	**95% CI**		***t***	***p***	***R*^2^**	**Δ *R*^2^**
			**LL**	**UL**				
(1)	Searching health information online	0.139	0.079	0.198	4.59	<0.001	0.53	
(2)	News	0.133	0.075	0.192	4.45	<0.001	0.082	0.029
(3)	Age	0.094	0.035	0.152	3.23	<0.001	0.097	0.015
(4)	Education	0.124	0.067	0.181	4.29	<0.001	0.106	0.009
(5)	Distress by information	0.137	0.075	0.199	4.35	<0.001	0.115	0.009
(6)	Information avoidance	−0.142	−0.205	−0.080	−4.46	<0.001	0.130	0.015
(7)	Physical health condition	0.086	0.027	0.144	2.88	0.004	0.136	0.006
(8)	Gender	−0.060	−0.117	−0.002	−2.05	0.041	0.139	0.003

*n = 1,055. CI, confidence interval; LL, lower limit; UL, upper limit.*

### Mediation Analysis With Information Avoidance

**FIGURE 2:**
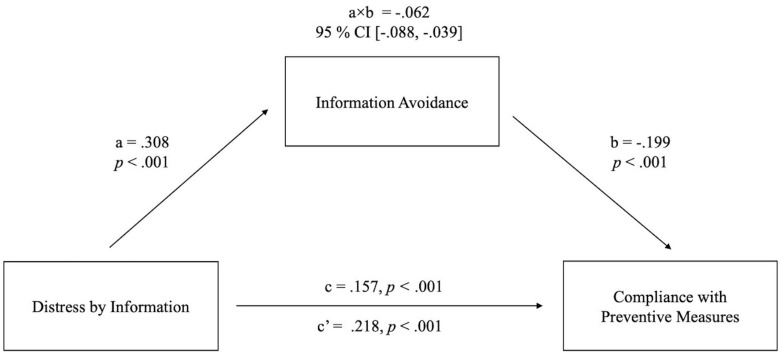
Schematic representation of the mediation model calculated with 5,000 bootstrap samples using PROCESS software. The pathway from distress by information to information avoidance (a) and then to compliance with preventive measures (b) represents the indirect effect of distress by information on compliance with preventive measures, mediated by information avoidance (referred to as a × b path). The path from distress by information to compliance with preventive measures (c’) shows the direct effect. The total effect of distress by information on compliance with preventive measures (c) is also shown on this path. Sociodemographic variables (age, gender, education), anxiety, and eHealth literacy were controlled for in this analysis.

Distress by information predicted better compliance with preventive measures and higher avoidance of information. Information avoidance, in turn, predicted worse compliance. Thus, we followed up on this in a mediation analysis (Model 4 in PROCESS; Hayes, 2020) to test whether information avoidance mediates an indirect negative effect of distress by information on compliance with preventive measures that runs counter to the overall positive effect. Results showed that the total effect of distress by information on compliance was positive, c path = 0.157, *p* < 0.001. This effect consisted of a direct positive effect of distress by information on compliance, c’ path = 0.218, *p* < 0.001, and a small indirect negative effect on compliance, mediated by avoidance of information, a × b path = −0.062, 95% CI (−0.088, −0.039). The mediation model is shown in Figure 2.

## Discussion

The ongoing COVID-19 pandemic obviously has a major impact on our emotions and our behavior. This study provides insights into the consequences of distress people experience from information about COVID-19 and information avoidance during this global health crisis. Overall, distress by information predicted better compliance. However, this was clearly diminished when distress led to information avoidance (avoidance hypothesis), which lessened compliance (compliance hypothesis). Both findings expand upon the growing body of literature on distress during the COVID-19 outbreak (Bao et al., 2020; Qiu et al., 2020; Rajkumar, 2020; Torales et al., 2020), by specifying the consequences of distress by information on information avoidance and compliance. Furthermore, our results underscore the critical role of trust in information sources and eHealth literacy. Higher trust was associated with less information avoidance, and individuals with higher eHealth literacy reported less distress by information and less avoidance of information.

Overall, distress was associated with better compliance with preventive measures and may thereby ultimately benefit crisis management. Most likely, this is because emotional salience typically increases attention and motivation. This is in line with previous findings on crisis behavior, which indicate that more anxious or worried individuals may be more compliant with preventive measures (Jones and Salathe, 2009; Rubin et al., 2010). However, when individuals respond to distress by avoiding information on COVID-19, this desirable effect on compliance is diminished. This corresponds with other findings showing that avoidance is a maladaptive strategy to reduce distress (Pittig et al., 2014). With respect to prevention, avoidance has been found to act as a barrier to preventive health behaviors (e.g., Cutler and Hodgson, 2003; Hightow et al., 2003; Howell and Shepperd, 2013a; Emanuel et al., 2015; Taber et al., 2015). Our findings highlight that information avoidance may be central to the negative consequences of information-related distress and may thereby interfere with crisis management.

Whether distress by information leads to avoidance is likely the consequence of personal coping style. Past research showed that individuals’ responses to threatening health information critically depend on their tendency to monitor or blunt threatening information (Miller, 1987, 1995; Williams-Piehota et al., 2005). Whereas *monitors* cope with distress by surveilling threatening information and taking appropriate measures, *blunters* are more easily overwhelmed by threatening information and avoid it (Williams-Piehota et al., 2005). In line with this, information avoidance correlated negatively with monitoring and positively with blunting in a previous study (Howell and Shepperd, 2016). It is, thus, understandable that in our sample, behaviors that are typical for monitoring (e.g., watching more news than before the crisis, searching health information on the internet) were the best predictors for higher compliance with preventive measures. This may inspire future studies on behavior in the COVID-19 pandemic to address coping styles.

Besides avoiding negative emotions and fighting overexposure to a particular topic (Sweeny et al., 2010; Barbour et al., 2012), research has shown that information avoidance can result from the feeling that there is nothing one can do to prevent negative consequences (Miles et al., 2008; Taber et al., 2015). This may also be the case with COVID-19, as information regarding the effectiveness of preventive measures has been contradictory or changed over time (e.g., withdrawn Ibuprofen warnings; Sodhi and Etminan, 2020; Torjesen, 2020). Such contradictions may irritate individuals and encourage information avoidance.

Reducing avoidance of information may be particularly important in long-term crisis management. After an initial period of mandatory restrictions, regulations were relaxed in order to circumvent higher economic costs. At the same time, the goal was to prevent the uncontrolled spread of the virus with high casualties. Introducing preventive measures on a regional level appears to be a promising approach to contain the virus (Bittihn et al., 2020). This requires timely and tailored communication from governments as well as high information attainment from the public. Moreover, missing out on important novel information (e.g., rising COVID-19 incidences in one’s area of residence) may have detrimental consequences.

Critically, the successful containment of the virus may be impeded if opinions shift and the public considers the restrictions and preventive measures to fight COVID-19 to be exaggerated. As major viral outbreaks often occur in waves, making it through the first wave without adverse consequences can provide individuals with a false sense of security (Khosravi, 2020). In line with this, levels of anxiety and acceptance of preventive measures declined after the contact restrictions were relaxed in Germany (Betsch et al., 2020a). Such changes in emotional salience may bias retrospective evaluation of the crisis, as individuals tend to rate events less aversive once the peak of anxiety has passed (Müller et al., 2019). In light of this, a continuous emphasis on the benefits of receiving information and the necessity of preventive behaviors is pivotal to crisis management (Betsch et al., 2020a).

From a clinical perspective, we are well aware that avoidance can be a rather stable behavioral pattern (Pittig et al., 2018) and rational approaches are sometimes not sufficient to alter such habitual behavior (Alpers, 2010; Helbig-Lang et al., 2014). However, past research suggests that contemplation is a promising technique to reduce information avoidance, and thereby, it may also foster better compliance with preventive measures. Contemplation refers to deliberately thinking about the consequences of obtaining information vs. not obtaining information. In general, this draws an individual’s attention to the long-term benefits of receiving information and reduces avoidance of information (Howell and Shepperd, 2013b). This could be advocated in media campaigns that encourage individuals to stay informed, by outlining the benefits of receiving information and the perils of information avoidance. Similarly, calls to “stay at home” or “flatten the curve” were effectively communicated through the media early in the COVID-19 crisis. Furthermore, health messages distributed in the media should be tailored to individuals’ information preferences and coping styles, as this increased preventive behaviors in other health domains (Williams-Piehota et al., 2005).

The media is an important tool to keep the public informed in times of crisis. This is corroborated by our findings, showing that the majority of people used a variety of information sources and consumed more news during the COVID-19 crisis than before the crisis. Interestingly, health authorities’ social media channels were one of the most commonly used information sources. Thus, social media may be a particularly direct medium to effectively communicate information to the public (Lachlan et al., 2016). Moreover, our findings suggest that many individuals feel that they can discriminate between reliable and unreliable content within one kind of medium. For instance, a substantial percentage of participants obtained news from authorities’ social media channels (73.2%), but a much smaller percentage of participants obtained information from user-generated content on social media (25.4%). Furthermore, participants rated the authorities’ social media channels as more trustworthy than user-generated content. This implies that individuals critically evaluated the origin of the health information that they received, which we interpret in terms of adequate eHealth literacy.

Finally, our results underscore the critical role of trust in information sources and adequate eHealth literacy in crisis management. Both higher trust in information sources and higher eHealth literacy predicted less distress by information and less avoidance of information. These results are in line with past findings, which demonstrate that trust benefits crisis management (Rubin et al., 2009) and that more health literate individuals experience lower psychological distress when facing a disease (Lin et al., 2019) and report less avoidance of information (Strekalova, 2016). This emphasizes that low eHealth literacy may also be an indirect threat to global public health management in the COVID-19 crisis. However, authorities (e.g., Robert-Koch-Institute, WHO) are already addressing this in measures, such as making high-quality information about COVID-19 available in simple language. Expanding this to other high-quality media coverage may be one way to fight the implications of low eHealth literacy and information avoidance at the same time.

## Limitations

Our findings need to be considered in light of several limitations. First, we conducted a cross-sectional survey that means that causal inferences are beyond the scope of our data. Consequently, the possible mechanisms of actions that we discuss need to be verified in future studies. Nevertheless, our results are an important first step and provide promising starting points for future research.

Second, our sample is not representative of the general population in Germany, as the data were collected online and the majority of the participants were female and highly educated. Obviously, this limits the generalizability of the findings. However, we expect that our findings regarding information avoidance and compliance may underestimate actual correlations in a representative sample. Because we distributed the link to the study in social media groups that shared information on COVID-19 (e.g., Facebook groups named “corona information” or “corona help”), our sample may have been particularly eager to seek information on COVID-19. Furthermore, both female gender and higher education predicted more compliance in our regression analyses. Consequently, this bias likely led to an underestimation of our effects. Future studies should aim for a more balanced sample and may employ different sampling methods.

Third, to our knowledge, there was no established scale to measure compliance during a pandemic at the time point of data collection. Thus, we assessed compliance with a self-generated scale, which was not yet well-validated. This too may have resulted in underestimated effects. Moreover, we relied on self-report data, which generally need to be interpreted cautiously. However, the anonymous format of our survey may have minimized demand characteristics.

Finally, following the conventions by Cohen (1988), the effects we detected are small to moderate. In particular, the effects regarding compliance with preventive measures are small, and the regression model on compliance explained less variance than our other regression models. However, in part, this may be because we collected data in an early phase of the COVID-19 crisis. In this time, levels of distress and risk perception were most pronounced (Betsch et al., 2020c). In accordance, compliance may have been particularly high in our sample. However, as compliance decreased in subsequent stages of the crisis (Betsch et al., 2020b), the repercussions of information avoidance may now be even more pronounced.

## Conclusion

In sum, the present findings show that experiencing distress by information about COVID-19 may influence compliance with preventive measures. While such distress may generally foster compliance, distress can also induce information avoidance, and this, in turn, lessens compliance with preventive measures. Thus, we consider information avoidance a maladaptive response to exacerbated distress. From a public health perspective, this may interfere with crisis management. As the adequate provision of information may be particularly important in sustained crisis management, measures to counteract information avoidance should be developed and implemented in a timely manner.

## Data Availability Statement

The data supporting the conclusions of this article have been deposited on MADATA (University of Mannheim) Research Data Repository (doi: 10.7801/345) and will be made available by the authors, without undue reservation, to any qualified researcher.

## Ethics Statement

This study was carried out in accordance with the recommendations of the ethics committee of the University of Mannheim. All participants received information about the study purpose and procedure and gave informed consent prior to participation. Participants who did not consent were not granted access to the online survey. As no personal data was collected, other than in the questionnaire, participants remained completely anonymous.

## Author Contributions

All authors contributed to research conceptualization and design. KUS and AKK implemented the questionnaire and analyzed the results. KUS drafted the manuscript and all authors contributed to reviewing and editing. GWA provided the resources.

## Conflict of Interest

The authors declare that the research was conducted in the absence of any commercial or financial relationships that could be construed as a potential conflict of interest.
