# Supplementary material for: Dealing With the COVID-19 Infodemic: Distress by Information, Information Avoidance, and Compliance With Preventive Measures
Source: Front Psychol. 2020 Nov 5;11:567905. doi: 10.3389/fpsyg.2020.567905 (PMC7674611; doi:10.3389/fpsyg.2020.567905)
Supplement: Supplementary file 1 [file Table_1.DOCX]

***Supplementary Material***

1. **Statistical Values of the Trust Ratings**

**Table S1**

*Comparison of Trust Ratings Provided by Participants who use (Users) or do not use (non-users) an Information Source*

|  |  | |  | |  | |  |  |  |
| --- | --- | --- | --- | --- | --- | --- | --- | --- | --- |
| **Information Sources** | | | **Users** | | **Non-Users** | |  |  |  |
|  |  | |  | |  | |  |  |  |
|  |  | |  | |  | |  |  |  |
|  |  | | ***n*** | ***M(SD)*** | ***n*** | ***M(SD)*** | ***t(df)*** | ***p*** | ***d*** |
|  |  | |  | |  | |  |  |  |
|  |  | |  | |  | |  |  |  |
| **Media Sources** | | |  | |  | |  |  |  |
|  | | |  | |  | |  |  |  |
|  | | News Channels’ Websites | 891 | 4.15 (0.83) | 168 | 3.41 (1.20) | 7.67 (198.27) | <.001 | 0.82 |
|  | | Internet Search Engines | 619 | 3.14 (0.75) | 440 | 2.61 (0.83) | 10.64 (887.46) | <.001 | 0.67 |
|  | | Social Media (Authorities’ Channels) | 775 | 4.08 (0.92) | 284 | 3.55 (1.14) | 7.14 (426.09) | <.001 | 0.54 |
|  | | Social Media (User Generated Content) | 269 | 2.63 (0.87) | 790 | 1.97 (0.89) | 10.70 (1057) | <.001 | 0.75 |
|  | | TV (Public) | 735 | 4.24 (0.81) | 324 | 3.49 (1.15) | 10.65 (470.55) | <.001 | 0.81 |
|  | | TV (Private) | 272 | 3.51 (0.99) | 787 | 2.62 (1.01) | 2.72 (1057) | <.001 | 0.86 |
|  | | Newspaper | 333 | 3.83 (0.76) | 726 | 3.39 (0.99) | 7.79 (818.98) | <.001 | 0.48 |
|  |  | |  |  |  |  |  |  |  |
|  |  | |  |  |  |  |  |  |  |
| **Other Sources** | | |  |  |  |  |  |  |  |
|  | | |  |  |  |  |  |  |  |
|  | | Primary Care Physician | 79 | 4.19 (0.80) | 980 | 3.87 (0.91) | 3.02 (1057) | .003 | 0.36 |
|  | | Friends & Family | 443 | 3.08 (0.79) | 616 | 2.56 (0.84) | 10.35 (983.18) | <.001 | 0.64 |
|  | | Health Authorities | 293 | 4.30 (0.85) | 766 | 4.11 (0.98) | 3.04 (1057) | .004 | 0.20 |
|  |  | |  |  |  |  |  |  |  |

*Note.* *N* = 1059. Trust Ratings were provided on a scale from 1 = not trustworthy to 5 = trustworthy. User) = Trust ratings provided by participants who report to use this source to obtain information of COVID-19; Non-Users = Trust ratings provided by participants who report not to use this source to obtain information of COVID-19.

1. **Factor Analysis for the Compliance Index**

The behaviors assessed in our compliance index include 1) staying at home, 2) following recommended hygiene regulations (washing hands regularly, cough and sneeze etiquette), 3) keeping an appropriate distance from other people, 4) wearing a face mask, 5) having in-person social contact, 6) going to a park or playground, 7) going to the gym, 8) going to a party, 9) going to a restaurant, 10) taking a trip,11) visiting family, 12) using public transportation, and 13) excessive purchases.

We ran an exploratory factor analysis using varimax rotation. The Kaiser-Meyer-Olkin measure was .866, and Bartlett’s test of sphericity was significant (χ2 (78) = 2648.84, *p* < .001. Also, the values in the diagonal of the anti-image matrix were >1. Furthermore, all but five items (staying at home, visiting family, excessive purchases, using public transport, wearing a face mask) had communalities >.4. Exact values are shown in **Table 2**.

Using principal component analysis, two factors were extracted with an eigenvalue greater 1. Factor loadings are shown in **Table 2.** Factor 1 explained 26.03% variance and included item 1, 5, 6, 7, 8, 9, 10, and 11 and showed adequate internal consistency (Cronbach's α = 0.79). Factor 2 explained 13.37% variance and included item 2, 3, 4, 12 and 13 and demonstrated inadequate internal consistency (Cronbach's α = 0.44). Although the internal consistency of the second factor was low, we induced all 13 items into our final index, as all items assess behaviors that are highly relevant in the ongoing COVID-19 pandemic.

**Table S2**

*Communalities and Factor Loadings of all Items in the Compliance Index*

|  | |  |  |  |
| --- | --- | --- | --- | --- |
| **Behavior** | | **Communalities** | **Factor 1** | **Factor 2** |
|  | |  |  |  |
|  |  |  |  |  |
|  | (1) Staying at home | .286 | .434 | .314 |
|  | (2) Hygiene Regulations | .448 |  | .656 |
|  | (3) Keeping distance | .413 |  | .582 |
|  | (4) Face Mask | .309 |  | .548 |
|  | (5) Social Contact* | .436 | .651 |  |
|  | (6) Park/ Playground* | .431 | .656 |  |
|  | (7) Gym* | .412 | .637 |  |
|  | (8) Party* | .566 | .774 |  |
|  | (9) Restaurants* | .625 | .747 |  |
|  | (10) Trip* | .429 | .632 |  |
|  | (11) Visit Family* | .283 | .471 |  |
|  | (12) Public Transport* | .238 |  | .433 |
|  | (13) Excessive Purchases* | .245 |  | .488 |

*Note.* *N* = 1059. Factor loadings <.3 are suppressed. All items marked with an asterisk (*) are inverted, thus participants were considered more compliant when they showed less of these behaviors. Items that are not marked are not inverted, thus participants were considered compliant when they showed more of these behaviors.

**Table S3**

*Percentages of Participants who Complied with Each Behavior in the Index*

|  | |  |
| --- | --- | --- |
| **Behaviors** | | **Compliers**  **(%)** |
|  | |  |
|  | |  |
| (1) | Staying at home | 92.4 |
| (2) | Following Hygiene Regulations | 87.2 |
| (3) | Keeping Distance | 92.3 |
| (4) | Wearing a Face Mask | 29.9 |
| (5) | Social Contact* | 90.9 |
| (6) | Going to the Park/ Playground* | 78.8 |
| (7) | Going to the Gym* | 75.4 |
| (8) | Going to a Party* | 89.6 |
| (9) | Going to a Restaurants* | 90.6 |
| (10) | Taking a Trip* | 84.2 |
| (11) | Visiting Family* | 76.6 |
| (12) | Public Transport* | 64.7 |
| (13) | Excessive Purchases* | 45.1 |
|  |  |  |

*Note.* *N* = 1059. All items marked with an asterisk (*) are inverted, thus participants were considered more compliant when they showed less of these behaviors. Items that are not marked are not inverted, thus participants were considered compliant when they showed more of these behaviors.

1. **Data Distribution and Outliers**

**Table S4**

*Means and Standard Deviations of Raw Values and Transformed Values of the Main Outcome Variables and Predictors*

|  | |  | | |  | | |  |  |
| --- | --- | --- | --- | --- | --- | --- | --- | --- | --- |
| **Measures** |  |  | | **Raw Values** | | |  | **Linearly Transformed Values** | |
|  |  |  | |  | | |  |  | |
|  | |  | | | | | |  | |
|  | | ***M*** | | | ***SD*** | | | ***M*** | ***SD*** |
|  | |  | | |  | | |  |  |
|  | |  | | |  | | |  |  |
| Distress | | 9.10 | | | 3.05 | | | 31.88 | 19.08 |
| Information Avoidance | | 28.27 | | | 11.39 | | | 35.69 | 21.08 |
| eHealth Literacy | | 33.77 | | | 5.02 | | | 80.53 | 15.69 |
| Anxiety | | 13.35 | | | 4.98 | | | 30.26 | 23.72 |
| Trust* | | 3.81 | | | 0.69 | | | 70.36 | 17.23 |
|  | |  | | |  | | |  |  |
|  | |  | | |  | | |  |  |
|  | |  | **Raw Values** | | |  | | **Log Transformed Values** | |
|  | |  |  | | |  | |  | |
|  | |  | | | | | |  | |
|  | | ***M*** | | | ***SD*** | | | ***M*** | ***SD*** |
|  | |  | | |  | | |  |  |
|  | |  | | |  | | |  |  |
| Compliance | | 9.97 | | | 2.49 | | | 1.42 | .59 |
|  | |  | | |  | | |  |  |

*Note.* *N* = 1059; *Trust, *n* = 1055, as participants used a different number of sources, we present the average trust rating of a used information source instead of the sum score. Linearly transformed values are transformed to a scale of 0-100. Log-transformed values were transformed using a reverse score log-transformation for left-skewed data.

**Figure S1**

*Boxplots Showing the Distribution of the Main Outcome Measures and Predictors*


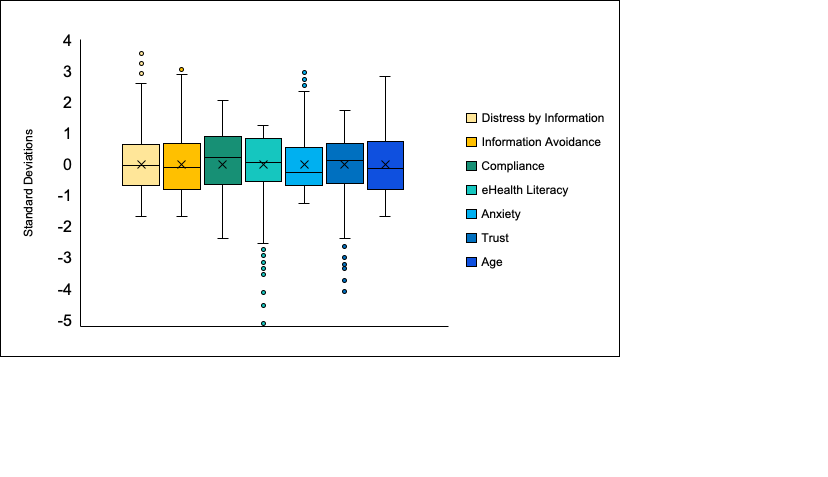


*Note.* *N* = 1059; Trust, *n* = 1055. The figure shows the distribution of the main measures in the analyses. Z-scores were used to compute the boxplots. X marks the mean and the line inside the box marks the median of the data. Whiskers are set to a maximal length of 1.5 times the interquartile range (IQR). Points exceeding this distance are shown as outliers. eHealth literacy and trust in information sources used were most affected by outliers.

1. **Analysis without Outliers**

We excluded all cases that were outliers (± 3 SD) on the measures for compliance, information avoidance, distress by information, trust in information sources used, eHealth literacy, generalized anxiety, and age. Overall 36 cases were excluded, and we repeated our main analyses with the remaining data (n = 1033). Overall, the gist of our findings did not change when outliers were removed. **Table S5** and **Table S6** provide the results of the regression analyses on information avoidance and compliance without outliers.

**Table S5**

*Summary of the Final Regression Model on Information Avoidance Excluding Outliers*

|  | |  |  |  |  |  |  |  |
| --- | --- | --- | --- | --- | --- | --- | --- | --- |
| **Step** | **Predictor** | ***ß*** | ***95% CI*** | | ***T*** | ***p*** | ***R²*** | ***ΔR²*** |
|  |  |  |  | |  |  |  |  |
|  |  |  |  | |  |  |  |  |
|  |  |  | ***LL*** | ***UL*** |  |  |  |  |
|  | |  |  |  |  |  |  |  |
|  | |  |  |  |  |  |  |  |
| (1) | Distress by Information | .336 | .269 | .403 | 9.84 | <.001 | .090 |  |
| (2) | Trust | -.163 | -.221 | -.106 | -5.56 | <.001 | .121 | .031 |
| (3) | Age | -.175 | -.231 | -.119 | -6.13 | <.001 | .143 | .022 |
| (4) | eHealth Literacy | -.135 | -.194 | -.076 | -4.5 | <.001 | .161 | .018 |
| (5) | Anxiety | -.139 | -.205 | -.073 | -4.12 | .003 | .173 | .012 |
|  |  |  |  |  |  |  |  |  |

*Note. n* = 1033. All outliers ± 3 SD were not included in this analysis. Exact statistical values have changed, but the same predictors reach significance. *CI* = confidence interval; *LL* = lower limit, *UL* = upper limit.

**Table S6**

*Summary of the Final Regression Model on Compliance Excluding Outliers*

|  |  |  |  |  |  |  |  |  |
| --- | --- | --- | --- | --- | --- | --- | --- | --- |
| **Step** | **Predictor** | ***ß*** | ***95% CI*** | | ***T*** | ***p*** | ***R²*** | ***ΔR²*** |
|  |  |  |  |  |  |  |  |  |
|  |  |  |  |  |  |  |  |  |
|  |  |  | ***LL*** | ***UL*** |  |  |  |  |
|  |  |  |  |  |  |  |  |  |
|  |  |  |  |  |  |  |  |  |
| (1) | Searching Health Information Online | .139 | .079 | .199 | 4.53 | <.001 | 0.48 |  |
| (2) | News | .129 | .070 | .189 | 4.28 | <.001 | .073 | .025 |
| (3) | Age | .095 | .036 | .155 | 3.15 | .002 | .089 | .016 |
| (4) | Education | .117 | .059 | .175 | 3.98 | <.001 | .097 | .008 |
| (5) | Distress by Information | .141 | .078 | .204 | 4.38 | <.001 | .106 | .009 |
| (6) | Information Avoidance | -.126 | -.190 | -.063 | -3.91 | <.001 | .119 | .014 |
| (7) | Physical Health Condition | .085 | .026 | .144 | 2.81 | .005 | .124 | .005 |
|  |  |  |  |  |  |  |  |  |

*Note. n* = 1033. All outliers ± 3 SD were not included in this analysis. Exact statistical values have changed, but the same predictors reach significance, except for gender, which no longer reached significance. *CI* = confidence interval; *LL* = lower limit, *UL* = upper limit.
